# Greater Perceived Physical Fatigability Is Associated with Lower Cognition: The Long Life Family Study

**DOI:** 10.1093/geroni/igaa057.2831

**Published:** 2020-12-16

**Authors:** Theresa Gmelin, Andrea Rosso, Stacy Andersen, Stephanie Cosentino, Mary Wojczynski, Kaare Christensen, Robert Boudreau, Nancy Glynn

**Affiliations:** 1 University of Pittsburgh, Pittsburgh, Pennsylvania, United States; 2 School of Public Health, University of Pittsburgh, Pittsburgh, Pennsylvania, United States; 3 Boston University School of Medicine, Boston, Massachusetts, United States; 4 Columbia University Medical Center, New York, New York, United States; 5 Washington University School of Medicine, St Louis, Missouri, United States; 6 University of Southern Denmark, Odense C, Syddanmark, Denmark

## Abstract

Greater perceived physical fatigability is associated with physical functional decline, but few studies have examined its relation with cognition. Adults ≥60 (mean±SD age 73.7±10.5, 54.7% female, 99.6% white) from the Long Life Family Study (n=2355) completed the Pittsburgh Fatigability Scale (PFS, 0-50, higher=greater fatigability) and a neurocognitive examination. Generalized estimating equations were used to account for family structure. Covariates included age, sex, field center, depressive symptoms (Center for Epidemiological Studies-Depression), education, and self-reported health. Each 1-point greater PFS was associated with lower: (1) global cognition (Mini-Mental Status Exam; β=-0.36,p<.0001), (2) verbal fluency (phonemic: β=-0.09,p=.029 and semantic: β=-0.14,p<.0001), (3) memory (Hopkins Verbal Learning Test-Revised: β=-0.06,p=.037), and (4) psychomotor speed (Digit Symbol Substitution Test: β=-0.10,p<.0001), after covariate adjustment. Greater perceived physical fatigability was significantly associated with lower memory and cognitive function in older adults, and may represent a promising new biomarker of biological aging reflecting declining brain reserve, resilience, and neurodegeneration.

